# Crosstalk between ubiquitination and other post-translational modifications in cardiovascular diseases

**DOI:** 10.1016/j.isci.2026.117606

**Published:** 2026-09-24

**Authors:** Yi Yan, Zhiqiang Li, Tingting Xiao

**Affiliations:** 1 National Key Clinical Specialty, Department of Cardiology, Zhongda Hospital, Southeast University, Nanjing 210009, China; 2 Department of General Surgery, the First Affiliated Hospital of Nanjing Medical University, Nanjing 210029, China; 3 Department of Cardiology, Tinghu People’s Hospital of Yancheng City, Yancheng 224000, China

**Keywords:** crosstalk, ubiquitination, post-translational modifications, cardiovascular diseases

## Abstract

Cardiovascular diseases (CVDs) remain a major cause of morbidity and mortality worldwide. Post-translational modifications (PTMs) are increasingly recognized as important regulators of cardiovascular pathophysiology, but how different PTMs interact in CVDs is still incompletely understood. This review focuses on the crosstalk between ubiquitination and other PTMs, including phosphorylation, SUMOylation, acetylation, methylation, O-GlcNAcylation, neddylation, and ADP-ribosylation/PARylation. We summarize representative cardiovascular mechanisms in which these modifications act competitively, cooperatively, or sequentially to influence protein stability, signaling, and function. We also discuss current limitations in establishing PTM interactions and highlight areas that require further investigation. A clearer understanding of these regulatory relationships may help identify disease-relevant PTM pathways and potential therapeutic targets in CVDs.

## Teaser

Disease; Molecular biology

## Introduction

Cardiovascular diseases (CVDs) continue to be the leading cause of mortality globally, with 18.6 million deaths attributed to them in 2017, as reported by the Global Burden of Disease (GBD) study.^1^ The total number of cardiovascular deaths has been estimated to have risen from 12.1 million in 1990 to 18.6 million in 2019, and this trend is projected to persist, with predictions suggesting that CVD-related deaths will surpass 23.6 million globally by 2030.^1^^,^^2^^,^^3^^,^^4^ The mortality and morbidity rates associated with CVD, which stem from various factors such as myocardial ischemia/reperfusion (I/R) injury, myocardial infarction (MI), cardiomyopathy, myocarditis, heart failure (HF), atherosclerosis, hypertension (HT), and ischemic stroke, underscore the need for comprehensive investigations to devise effective and prompt strategies to mitigate these challenges.^5^^,^^6^ However, the molecular mechanisms that underlie the development of CVDs remain largely elusive and not fully comprehended.

CVDs arise from both genetic susceptibilities (e.g., familial cardiomyopathies) and environmental or acquired factors (e.g., high glucose, HT, and ischemia-reperfusion), and post-translational modifications (PTMs) crosstalk—particularly that involving ubiquitination—is critically involved across this entire disease spectrum. CVDs are frequently accompanied by abnormal protein function at the cellular level.^7^ This has a profound impact on the homeostasis and disease progression of the cardiovascular system, as proteins are regulated through their stability, activity, and subcellular localization.^8^^,^^9^ PTMs are important regulatory mechanisms that modify proteins after translation and alter their biochemical properties. It is estimated that PTMs influence approximately 50%–90% of all human proteins, and their intricate functional regulation is primarily mediated by diverse PTMs.^10^ Each type of PTM involves specific enzymes that mediate the modification and de-modification processes, ensuring a dynamic balance.^8^ However, dysregulation of these enzymes can contribute to various pathologies, driving the development of diseases. Recent technological advancements have facilitated the discovery of several dysregulated enzymes that contribute to imbalanced PTMs in various diseases, including CVDs.^11^^,^^12^^,^^13^^,^^14^^,^^15^ In CVDs, PTMs play a pivotal role in regulating various cellular processes such as DNA damage repair, signal transduction, immune responses, metabolic reprogramming, cell proliferation, cell cycle regulation, angiogenesis, malignant transformation, cell epithelial-to-mesenchymal transition (EedMT), invasion, autophagy, and apoptosis.^9^^,^^14^^,^^15^

Ubiquitination, initially identified as a regulator of cellular protein turnover and homeostasis four decades ago, has since expanded its roles significantly.^16^^,^^17^ This process involves the enzymatic conjugation of free ubiquitin (Ub), a small 76-amino-acid protein, to the lysine (K) residue of target proteins.^18^ The attachment of mono-Ub or poly-Ub chains is orchestrated by a three-enzyme cascade comprising E1 (Ub-activating enzyme, UBA), E2 (Ub-conjugating enzyme, UBC), and E3 (Ub ligase). Following the initial Ub attachment, this enzymatic cascade can be reiterated, leading to the formation of a poly-Ub chain attached to the substrate through the sequential addition of Ub moieties to the lysine residue of the preceding Ub molecule (Figure 1A). Notably, the amino acid sequence of Ub is universally conserved across vertebrates and higher plants, with minimal variations (two or three residues) observed among animal, plant, and fungal Ubs.^19^ In contrast, deubiquitinases (DUBs) remove Ub, thereby recycling Ub, rescuing selected substrates from degradation, and modulating signaling pathways.^20^^,^^21^

**Figure 1:**
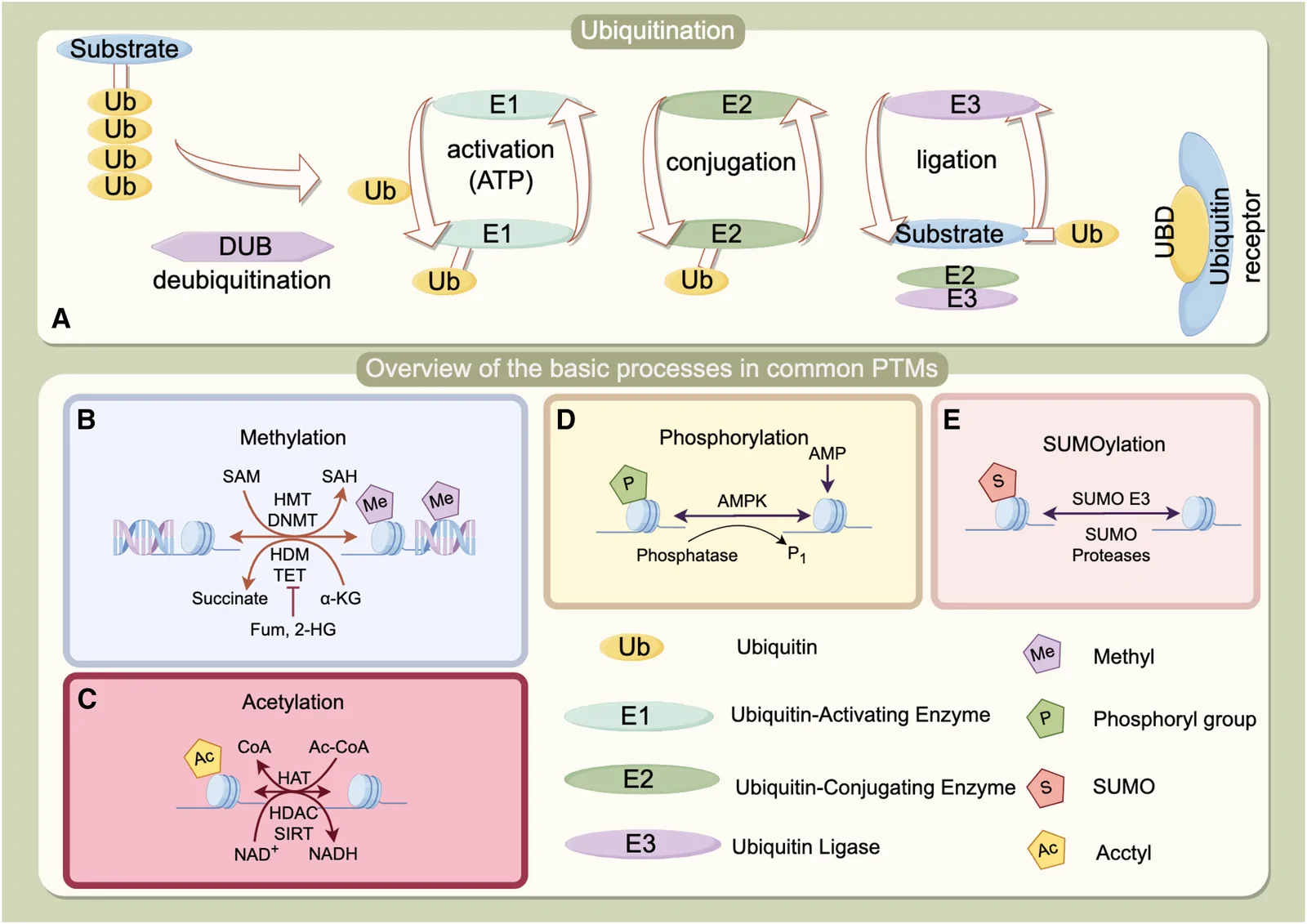
Overview of the basic processes in ubiquitination and common PTMs (A) Ubiquitination is a three-step enzymatic process where E3 ligases determine substrate specificity. DUBs remove ubiquitin from ubiquitinated substrates. (B) Methylation adds methyl groups to proteins. SAM (S-adenosyl methionine) serves as a methyl donor, and various methyltransferases (HMT, DNMT, and TET) catalyze the transfer of methyl groups to proteins. The process can also involve the conversion of succinate to fumarate and 2-HG, which can be influenced by α-KG. (C) The lysine residues of substrate can be acetylated by HAT with the help of acetyl-CoA, while acetylated substrate might be deacetylated via HDACs or SIRTs. (D) Phosphorylation is regulated by kinases, while phosphatases control dephosphorylation. (E) SUMOylation involves adding SUMO to proteins through activation by E1, conjugation by E2, and ligation by E3. SUMO proteases can remove SUMO groups, reversing the modification. PTMs, post-translational modifications; DUBs, deubiquitinating enzymes; SAM, S-adenosyl methionine; HMT, histone methyltransferase; DNMT, DNA methyltransferase; TET, ten-eleven translocation; 2-HG, 2-hydroxyglutarate; α-KG, α-ketoglutarate; HAT, histone acetyltransferase; acetyl-CoA, acetyl coenzyme A; HDACs, histone deacetylases; SIRTs, sirtuins; SUMO, small ubiquitin-like modifier.

Ubiquitination plays a pivotal role in the progression of CVDs, influencing various cellular processes such as the functionality of cardiac membrane channels and receptors, and the apoptosis of cardiomyocyte and vascular smooth muscle cells.^22^^,^^23^^,^^24^ Research indicates that targeting specific E3 Ub ligases through compounds or inhibitors could offer therapeutic benefits for CVDs, suggesting that modulating ubiquitination may be a promising strategy.^25^^,^^26^ However, it is crucial to recognize that CVDs are regulated by a complex interplay of signaling pathways involving multiple PTMs, rather than just one. During disease progression, multiple PTMs or the same PTM at different sites can simultaneously or sequentially occur on a substrate, contributing to distinct signaling outcomes. Consequently, elucidating the crosstalk between different PTMs is essential for unraveling the underlying molecular mechanisms and developing precise therapeutic interventions. To date, ubiquitination has been shown to interact with various PTMs, including phosphorylation, SUMOylation, acetylation, and methylation, in CVDs. In this review, we focus on the crosstalk between ubiquitination and other PTMs and discuss its molecular basis and relevance to CVDs.

## Crosstalk between PTMs

PTMs are essential for cellular regulation and involve complex interactions between catalytic machineries and substrate proteins. These modifications can crosstalk through the enzymes that modify each other, leading to positive or negative regulation of their activities.^27^^,^^28^^,^^29^^,^^30^ Many PTMs, such as ubiquitination, SUMOylation, phosphorylation, acetylation, and methylation, are reversible, which adds to their dynamic nature and crosstalk potential^28^^,^^29^^,^^31^ (Figure 1). Crosstalk can also occur when different PTMs target the same substrate protein, either modifying the same amino acid residues at different times or different residues altogether, resulting in synergistic or antagonistic effects on the protein’s function.^32^^,^^33^^,^^34^

The interplay among PTMs is characterized by competitive modification scenarios where multiple enzymes compete for the same protein sites, leading to shifts in modification precedence. Additionally, the introduction of one modification can trigger or inhibit subsequent modifications, creating positive or negative regulatory cascades.^32^^,^^33^ Synergistic effects are also observed when multiple PTMs act on a single protein, significantly altering its activity and function through cumulative mechanisms.^35^^,^^36^ In CVDs, PTM crosstalk can occur either through interactions between modifying enzymes or through multiple modifications of the same substrate.^34^ These interactions may alter protein activity, localization, stability, or degradation, providing several layers of regulation beyond the effects of individual PTMs.

## Phosphorylation and ubiquitination in cardiovascular diseases

Phosphorylation is the kinase-catalyzed addition of phosphate groups to proteins (Figure 1D) and regulates protein activity, stability, and localization.^37^^,^^38^ In the cardiovascular system, phosphorylation governs signal transduction, ion channel function, and cell survival.^38^ Its crosstalk with ubiquitination can alter both signaling activity and protein turnover. We discuss this relationship in three mechanistic categories: phosphorylation that inhibits ubiquitination, phosphorylation that primes proteins for ubiquitination and degradation, and ubiquitination that regulates phosphorylation signaling.

### Phosphorylation inhibits ubiquitination to stabilize proteins

Phosphorylation can antagonize ubiquitination and thereby protect selected proteins from proteasomal degradation. One example is aortic dissection (AD), a condition where blood flows into the aortic wall through an intimal tear, creating a false lumen.^39^^,^^40^ The mitogen-activated protein kinase (MAPK) cascade is a central pro-inflammatory signaling pathway; its sustained activation promotes vascular smooth muscle cell dysfunction and aortic wall degradation, driving aortic dissection. Bestrophin3 (Best3) is a calcium-activated chloride channel protein expressed in vascular smooth muscle cells (VSMCs). A causal role for Best3 deficiency in aortic dissection was established by Zhang et al., who demonstrated that Best3 deficiency in VSMCs leads to activation of the upstream kinase MEKK2/3. Specifically, Best3 loss leads to enhanced phosphorylation of MEKK2 Ser153 and MEKK3 Ser61 (the specific kinase responsible for these phosphorylation events was not identified in the original report). This phosphorylation event does not directly regulate Ub degradation; rather, it stabilizes MEKK2/3 by inhibiting its recognition by the Ub-proteasome system, thereby increasing MEKK2/3 protein levels. The stabilized and activated MEKK2/3 then phosphorylates downstream MKKs and MAPKs, culminating in sustained MAPK signaling. This cascade promotes VSMC phenotypic switching and extracellular matrix degradation directly leads to the development of aortic dissection (Figure 2A).^39^

**Figure 2:**
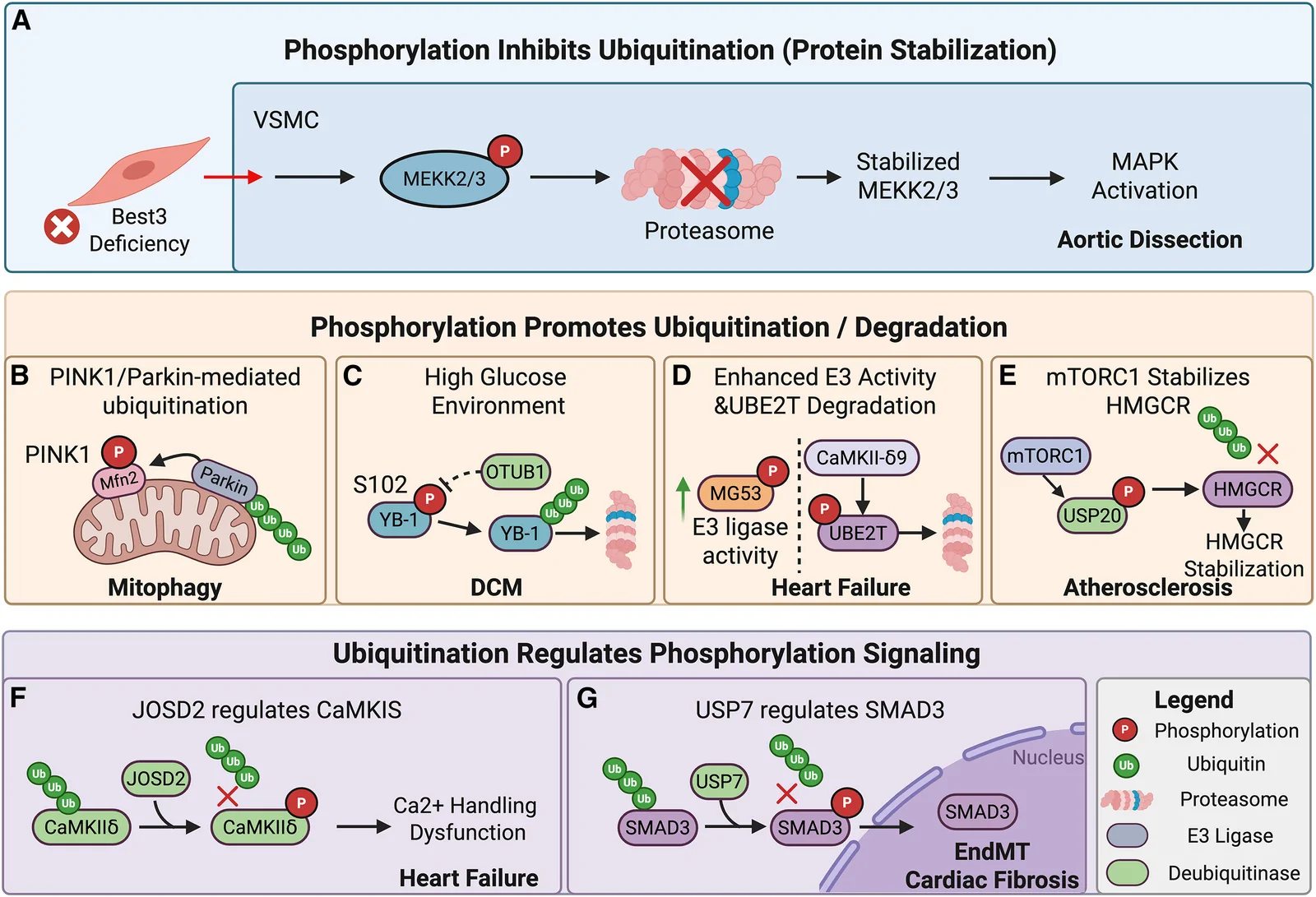
Mechanistic crosstalk between phosphorylation and ubiquitination in cardiovascular diseases This figure illustrates three major modes of crosstalk using specific cardiovascular disease exemplars. (A) Phosphorylation inhibits ubiquitination to stabilize proteins. In aortic dissection (AD), bestrophin 3 (Best3) deficiency in vascular smooth muscle cells (VSMCs) enhances phosphorylation of MEKK2 at Ser153 and MEKK3 at Ser61 (mitogen-activated protein kinase 2/3). This phosphorylation stabilizes MEKK2/3 by blocking their recognition by the ubiquitin-proteasome system, leading to sustained activation of downstream MKKs (MAPK kinases) and MAPKs (mitogen-activated protein kinases), ultimately promoting VSMC dysfunction and aortic wall degradation. (B–E) Phosphorylation promotes ubiquitination and degradation. (B) In cardiomyocytes, PTEN-induced kinase 1 (PINK1) accumulates on damaged mitochondria and phosphorylates mitofusin 2 (Mfn2), recruiting the E3 ubiquitin ligase Parkin to ubiquitinate Mfn2 and other mitochondrial proteins, thereby tagging damaged mitochondria for autophagic clearance (mitophagy). (C) In diabetic cardiomyopathy (DCM), high glucose activates ERK/RSK signaling (extracellular signal-regulated kinase/ribosomal S6 kinase), leading to phosphorylation of Y-box binding protein-1 (YB-1) at Ser102. This reduces YB-1 binding to the deubiquitinase OTUB1 (otubain-1), destabilizing YB-1 and aggravating diabetic heart injury. (D) In myocardial injury and heart failure, phosphorylation of MG53 at Ser255 enhances its E3 ubiquitin ligase activity, promoting ubiquitination of target proteins; similarly, CaMKII-δ9 (Ca^2+^/calmodulin-dependent protein kinase II δ9) phosphorylates ubiquitin-conjugating enzyme E2T (UBE2T), leading to its proteasomal degradation and disrupting DNA repair. (E) In atherosclerosis, mTORC1 (mammalian target of rapamycin complex 1) phosphorylates the deubiquitinase USP20 (ubiquitin-specific protease 20), which stabilizes HMG-CoA reductase and increases lipid biosynthesis. (F and G) Ubiquitination regulates phosphorylation signaling. (F) The deubiquitinase JOSD2 (josephin domain-containing protein 2) hydrolyzes K63-linked polyubiquitin chains on CaMKIIδ, thereby increasing CaMKIIδ phosphorylation and causing calcium mishandling, hypertrophy, and fibrosis. (G) USP7 (ubiquitin-specific protease 7) removes K63 ubiquitin chains from SMAD3, stabilizing SMAD3 and promoting its phosphorylation and nuclear translocation, which aggravates endothelial-to-mesenchymal transition (EndMT) and cardiac fibrosis. AD, aortic dissection; Best3, bestrophin 3; CaMKII, Ca^2+^/calmodulin-dependent protein kinase II; DCM, diabetic cardiomyopathy; EndMT, endothelial-to-mesenchymal transition; ERK, extracellular signal-regulated kinase; JOSD2, josephin domain-containing protein 2; MAPK, mitogen-activated protein kinase; MEKK2/3, mitogen-activated protein kinase 2/3; Mfn2, mitofusin 2; mTORC1, mammalian target of rapamycin complex 1; OTUB1, otubain-1; PINK1, PTEN-induced kinase 1; RSK, ribosomal S6 kinase; SMAD3, mothers against decapentaplegic homolog 3; UBE2T, ubiquitin-conjugating enzyme E2T; USP7/20, ubiquitin-specific protease 7/20; VSMC, vascular smooth muscle cell; YB-1, Y-box binding protein-1.

### Phosphorylation primes proteins for ubiquitination and degradation

Conversely, phosphorylation can act as an “eat-me” signal that promotes ubiquitination and subsequent proteasomal or autophagic degradation. This may occur through phosphorylation-induced conformational changes that facilitate recognition by E3 Ub ligases.

#### Phosphorylation-dependent degradation in heart disease

In failing hearts, small proline-rich protein 2B (SPRR2B) is phosphorylated, which enhances its recognition by the deubiquitinase USP7. Rather than promoting degradation, USP7 binding attenuates SPRR2B ubiquitination, thereby stabilizing SPRR2B and accelerating cell cycle progression in cardiac fibroblasts.^41^ This example illustrates that phosphorylation can also modulate deubiquitinase activity.

#### Mitophagy and mitochondrial quality control

In cardiomyocytes, PTEN-induced kinase 1 (PINK1) accumulates on damaged mitochondria and phosphorylates mitofusin 2 (Mfn2). This phosphorylation event serves as a receptor that recruits the E3 Ub ligase Parkin to the outer mitochondrial membrane. Parkin then ubiquitinates Mfn2 and other mitochondrial proteins, tagging the damaged organelle for autophagic clearance (mitophagy), thereby preserving mitochondrial integrity and preventing cardiomyocyte death (Figure 2B).^42^

#### Regulation of deubiquitinase-substrate interactions

Otubain 1 (OTUB1) is a deubiquitinase that normally binds to Y-box binding protein-1 (YB-1) and prevents its ubiquitination and degradation. YB-1 is a multifunctional transcription and translation factor. In a high-glucose environment mimicking diabetic cardiomyopathy (DCM), ERK/RSK signaling is activated, leading to phosphorylation of YB-1 at Ser102. This phosphorylation reduces YB-1’s interaction with OTUB1, destabilizing YB-1. Inhibition of the ERK pathway with U0126 blocks RSK-mediated YB-1 phosphorylation, restores YB-1-OTUB1 binding, and maintains YB-1 protein levels in the diabetic heart (Figure 2C).^43^

#### Modulation of E3 ligase activity and localization

The activity of some E3 Ub ligases is directly regulated by phosphorylation. For example, MG53 is phosphorylated at Ser255 after myocardial injury, which enhances its E3 ligase activity and promotes ubiquitination of target proteins (Figure 2D).^44^ Similarly, CaMKII-δ9 promotes the development of cardiomyopathy by disrupting the UBE2T-dependent DNA repair pathway. Specifically, CaMKII-δ9, the most predominant CaMKII-δ isoform in the heart, directly binds to and phosphorylates UBC E2T (UBE2T), resulting in proteasome-dependent degradation of UBE2T. This degradation disrupts the UBE2T-dependent DNA repair, particularly the DNA repair function of the Fanconi anemia (FA) pathway, leading to the accumulation of myocardial cell DNA damage and genomic instability, ultimately resulting in myocardial cell death and HF (Figure 2D).^45^

Phosphorylation can also influence the subcellular localization of ubiquitination machinery. Under insulin stimulation, protein kinase B (AKT) phosphorylates the E3 ligase TRIM24, stimulating its translocation from the nucleus to the cytoplasm, where it ubiquitinates downstream substrates.^46^ Additionally, AMPKα2 (AMP-activated protein kinase α 2) plays a crucial role in the heart by enhancing mitochondrial autophagy to protect the heart and prevent the development of HF. AMPKα2 enhances the PINK1-Parkin-SQSTM1 (sequestosome-1) signaling pathway by directly interacting with the Ser495 site of phosphorylated PINK1, promoting ubiquitination and clearance of damaged mitochondria.^47^

#### Phosphorylation of deubiquitinases

Finally, phosphorylation can also regulate ubiquitination indirectly by altering the activity of DUBs, which remove Ub from target proteins. Atherosclerotic CVDs are closely related to the increase of low-density lipoprotein cholesterol. Under normal dietary conditions, glucose and insulin activate the mammalian target of rapamycin 1 (mTORC1), which in turn phosphorylates the deubiquitinase USP20. USP20 subsequently stabilizes HMG-CoA reductase, increasing lipid biosynthesis (Figure 2E).^48^

### Ubiquitination regulates phosphorylation signaling

Although ubiquitination is often associated with protein degradation, it can also directly influence phosphorylation events.

#### Ubiquitination affecting phosphorylation-site accessibility

For example, in HF, JOSD2 (josephin domain-containing protein 2), as a deubiquitination enzyme, plays an important role in cardiomyocytes. JOSD2 directly hydrolyzes the K63-linked polyubiquitin chains on CaMKIIδ, thereby increasing the phosphorylation of CaMKIIδ and resulting in calcium mishandling, hypertrophy, and fibrosis in cardiomyocytes (Figure 2F).^49^ Conversely, USP7 promotes SMAD3 deubiquitination to maintain the stability of SMAD3 by removing the K63 Ub chain and preventing the degradation of SMAD3 by proteasomal process. USP7 also promotes SMAD3 phosphorylation and nuclear translocation, thereby aggravating EndMT and cardiac fibrosis (Figure 2G).^50^

#### Ubiquitination targeting kinases or signaling adaptors

In hypertrophy-related HF, TRIM16 interacts with PRDX1 and inhibits its phosphorylation. TRIM16-blocked PRDX1 phosphorylation was largely dependent on a direct interaction between TRIM16 and SRC and the resultant SRC ubiquitinational degradation. This results in reduced phosphorylation of the downstream target PRDX1, thereby suppressing pathological cardiac hypertrophy.^51^ Similarly, OTUD1 promotes pathological cardiac remodeling and HF by targeting STAT3 in myocardial cells. Mechanistically, OTUD1 binds to the SH2 domain of STAT3, leading to the deubiquitination of STAT3. Cysteine at position 320 of OTUD1 promotes phosphorylation and nuclear translocation of STAT3 through K63 deubiquitination, thereby increasing STAT3 activity and inducing myocardial cell inflammation, fibrosis, and hypertrophy.^14^

The reciprocal regulation of phosphorylation and ubiquitination also provides potential therapeutic entry points. Modulating selected kinases, phosphatases, E3 ligases, or DUBs may alter disease-relevant signaling or protein stability, although the specificity and context of such interventions will be critical.

## SUMOylation and ubiquitination in cardiovascular diseases

SUMOylation is the covalent attachment of small Ub-like modifier (SUMO) proteins to lysine residues (Figure 1E).^52^^,^^53^^,^^54^ Unlike ubiquitination, SUMOylation does not directly target proteins for degradation but regulates transcription, DNA repair, and cell cycle.^55^ UBC9 is an E2 enzyme that undergoes SUMOylation during embryonic development and interacts with the SUMO pathway to regulate cardiac development.^56^^,^^57^^,^^58^ These features provide several points at which SUMOylation can intersect with ubiquitination in CVDs.

SUMOylation and ubiquitination can coordinately regulate protein stability and function. SENP3 (sentrin/SUMO-specific protease 3) is an SUMO protease involved in maintaining SUMO homeostasis.^59^^,^^60^^,^^61^ Recent research indicates that SENP3 plays a pivotal role in cardiac development, particularly by regulating the Nodal signaling pathway, which influences the left-right asymmetric development of the heart and the aging of myocardial cells.^62^ During cardiac development, a dynamic balance between SUMOylation and deSUMOylation is essential for maintaining cardiac development and function.^52^^,^^63^ Studies have revealed that in mouse models with SENP3 knockout, the knockout results in embryonic death on day 8.5 of embryonic development, with fatal embryos exhibiting neural tube defects and asymmetric left-right cardiac looping abnormalities. At the cellular level, knockdown of SENP3 significantly inhibits cell growth, promotes apoptosis and aging, whereas overexpression of SENP3 has the opposite effect. Further molecular biology experiments demonstrate that SENP3 regulates the stability of Nodal protein through deSUMOylation. Additionally, Nodal becomes highly SUMOylated following SENP3 knockout, and SUMOylation inhibits the ubiquitination and Ub-proteasome degradation pathway of Nodal.^62^ However, when these proteins are subjected to abnormal ubiquitination modifications, they may be rapidly degraded, leading to impaired heart function. Therefore, the balance between SUMOylation and ubiquitination is crucial for maintaining the stability and function of cardiovascular-related proteins.

In addition, since both SUMO and Ub attach to the lysine (K) residues of the substrate protein, these two PTMs may compete with each other. Meanwhile, a significant number of substrate proteins that are co-modified by both SUMOylation and ubiquitination have been identified.^64^^,^^65^^,^^66^ SERCA2a is a crucial calcium pump in cardiac cells, regulating intracellular calcium levels to control heart contraction and relaxation.^67^^,^^68^ Dysregulated calcium handling is a hallmark of HF, leading to reduced contractility and arrhythmias, which is why SERCA2a is a critical therapeutic target. Dysfunction of SERCA2a is linked to CVDs, particularly HF, affecting cardiac performance and calcium homeostasis.^67^ On the one hand, SERCA2a is SUMOylated at lysines 480 and 585, and this SUMOylation is essential for maintaining the ATPase activity and stability of SERCA2a in both mouse and human cells.^69^ Meanwhile, in cardiomyocytes, SERCA2a can also be deSUMOylated by SENP2.^70^ Regulating SUMOylation offers a novel therapeutic strategy for treating HF.^69^^,^^71^ On the other hand, SERCA2a can also be deubiquitinated by deubiquitinases USP25 or JOSD2, thereby inhibiting cardiac hypertrophy and ameliorating the adverse prognosis of HF.^21^^,^^72^ SERCA2a is modified by K48-linked deubiquitination and SUMOylation. These two modifications occur on different lysine residues of SERCA2a, namely K169 and K480/585.^69^^,^^72^ It is worth noting that SERCA2a is not a single instance, and Connexin43 (Cx43) can also be ubiquitinated and SUMOylated.^73^^,^^74^^,^^75^ In CVDs, the imbalance of these two modification mechanisms may lead to excessive apoptosis or abnormal proliferation of cardiovascular cells, thereby exacerbating the disease process.

## Acetylation and ubiquitination in cardiovascular diseases

Recent research has highlighted the importance of protein acetylation in CVDs.^76^^,^^77^ Acetylation is the addition of acetyl groups to lysine residues, catalyzed by histone acetyltransferases (HATs) and reversed by histone deacetylases (HDACs) (Figure 1C).^78^ Acetylation modification can alter the structure, charge, and stability of proteins, thereby affecting their biological functions.^78^ It regulates vascular tone, cardiac rhythm, and structural integrity.^79^^,^^80^^,^^81^^,^^82^^,^^83^ The reversible nature of lysine acetylation, in contrast to the irreversible N-terminal acetylation, allows for dynamic responses to physiological cues and pathological stimuli.^84^^,^^85^ Studies have underscored the therapeutic potential of modulating acetylation, with specific lysine deacetylase inhibitors demonstrating cardioprotective and vasculoprotective effects.^84^ These findings suggest that targeted acetylation may offer a novel avenue for the development of therapeutics in the treatment and prevention of CVDs.^77^^,^^83^^,^^84^^,^^86^^,^^87^ Although direct evidence for acetylation-ubiquitination crosstalk remains limited, their potential cooperative and antagonistic effects can be inferred from studies of their respective roles in CVDs.

Acetylation and ubiquitination may have a synergistic effect in regulating protein stability in CVDs. On the one hand, acetylation modification can indirectly regulate the degradation rate of proteins, affecting their ability to be recognized by ubiquitin-proteasome pathway. For example, in cardiomyocytes, long-term lipid overload triggers acetylation of Mfn2 at the K243 site, further inducing its ubiquitination degradation. The ubiquitination degradation of Mfn2 leads to a decrease in its content, causing myocardial mitochondrial lipid droplet separation and lipid utilization disorders, ultimately resulting in a transition of cardiac function from compensatory to decompensated.^88^ Additionally, after heart injury, sirtuin 6 (SIRT6) activation in macrophages was found to be associated with deacetylation of STING, and this deacetylation correlated with reduced STING ubiquitination and increased STING protein stability. Immunoprecipitation experiments in Raw264.7 cells showed that SIRT6 inhibitor treatment increased STING acetylation and correspondingly increased STING ubiquitination levels.^89^ These correlative data suggest a potential modulatory role for SIRT6 in STING stability, but direct causality remains to be established *in vivo*. There are also studies indicating that HDAC3 inhibits the ubiquitination-mediated degradation of DNA methyltransferase 1 (DNMT1) through deacetylation, thereby suppressing the expression of Src homology domain 2-containing tyrosine phosphatase-1 (SHP-1) and ultimately promoting the development of HF.^90^ Myocardiac ischemia reperfusion injury (MIRI) is involved in the pathogenesis of various CVDs.^91^ Lysine acetyltransferase 5 (KAT5) regulates the transcription of U-box-containing protein 1 (STUB1) through acetylation, leading to ubiquitination and degradation of LATS 2, activating the YAP/β-catenin pathway.^92^ These studies all indicate a synergistic effect between acetylation and ubiquitination modifications. In addition, ubiquitination can also affect the occurrence of acetylation. WWP1 catalyzes the degradation of Kruppel-like factor 15 (KLF15) through K48-linked polyubiquitination, leading to excessive myocardial cell inflammation after MI. The degradation of KLF15 mediated by WWP1 contributes to upregulation of p65 acetylation and activation of MAPK inflammatory signaling in ischemic myocardium and hypoxic-treated cardiomyocytes.^26^ On the other hand, there may be direct interactions between certain deacetylases (such as HDACs) and Ub ligases, jointly regulating the modification state and stability of specific proteins. As mentioned in the previous section, SUMOylation and deubiquitination occur on different lysine residues of SERCA2a, namely K169 and K480/585. It is worth noting that SERCA2a is also acetylated at K492.^79^ This synergistic effect may be involved in regulating the SERCA2a activity in hearts.

Acetylation and ubiquitination may also exert antagonistic effects in CVDs. For example, cystathionine γ-lyase (CSEγ) is a hydrogen sulfide (H2S)-producing enzyme, which can mediate vasodilation and help alleviate HT.^93^^,^^94^ The selective HDAC6 inhibitor tubastatin A (TubA) significantly increased the protein stability of CSEγ and the acetylation level at K73 site.^95^^,^^96^^,^^97^^,^^98^ The ubiquitination and degradation of CSEγ induced by AngII were inhibited by TubA, indicating that AngII promotes HDAC6-dependent deacetylation of CSEγ at the K73 site, leading to Ub-mediated protein degradation, which is the basis of AngII-induced HT.^98^ This competitive relationship may affect the modification status and stability of CSEγ, thereby regulating their role in CVDs. In cardiomyocytes, HDAC6 inhibits the acetylation of human ether-a-go-go-related gene (hERG), thereby suppressing ubiquitination and leading to hERG stability.^99^ In some cases, acetylation and ubiquitination may also compete for the same substrate protein, resulting in antagonistic effects. Because both acetylation and ubiquitination can modify lysine residues, they may compete for the same site on a substrate. Poly(ADP ribose) polymerase 1 (PARP1) plays an important regulatory role in CVDs.^100^^,^^101^ PARP1 is a DNA repair enzyme whose excessive activation in CVDs depletes nicotinamide adenine dinucleotide (NAD+) and ATP, leading to cell dysfunction and death. It also promotes vascular inflammation, smooth muscle cell proliferation, and adverse remodeling, making it a key contributor to atherosclerosis, cardiac hypertrophy, and ischemia-reperfusion injury. Deacetylase SIRT2 interacts with PARP1 in the PARP-A-helic domain and deacetylates the K249 residue of PARP1. In addition, SIRT2 promotes ubiquitination of the K249 residue of PARP1 by mobilizing E3 Ub ligase WW domain containing protein 2 (WWP2), leading to proteasome-mediated degradation of PARP1.^23^^,^^102^ Knocking out SIRT2 in mice and cells increases PARP1 acetylation and decreases PARP1 ubiquitination, which in turn exacerbates oxidative stress-induced vascular damage and remodeling.^102^ Therefore, competitive binding of acetylation and ubiquitination at the same lysine site of PARP1 was revealed.

In addition to obvious competitive or cooperative relationships, sequential modifications of ubiquitination and acetylation in a pathway can also result in vastly different outcomes. EndMT plays a crucial role in promoting myocardial fibrosis and exacerbating cardiac dysfunction.^103^^,^^104^ When EndMT occurs, SIRT1 (NAD^+^-dependent deacetylase) can be degraded by ubiquitination, leading to reduced nuclear translocation of SIRT1 and inability to bind to Notch1 (NICD), resulting in the inability of acetylated NICD to degrade.^105^ Dapagliflozin (DAPA), as a sodium glucose junction transporter 2 (SGLT-2) inhibitor, can degrade NICD by inhibiting the phosphorylation and ubiquitination of SIRT1, ultimately leading to deacetylation.^105^^,^^106^ These findings provide new insights into the potential mechanism of DAPA in the treatment of non diabetes HF.

## Protein methylation and ubiquitination in cardiovascular diseases

Methylation adds methyl groups to lysine or arginine residues, primarily regulating gene expression (Figure 1B).^107^ The specificity of methylation modification depends on the type of methyltransferase and the position of substrate amino acids, and different methylation patterns can affect the activation or inhibition of gene expression.^108^^,^^109^ In the cardiovascular system, methylation patterns on histones can lead to the occurrence of many types of CVDs, such as congenital heart disease (CHD).^110^ Dysregulation of methylation, particularly the accumulation of asymmetric dimethylarginine (ADMA), has been identified as a risk factor for CVDs, potentially impairing endothelial function and contributing to atherosclerotic processes.^109^^,^^111^ Moreover, the role of protein arginine methyltransferases (PRMTs) in vascular and neuronal function suggests that these enzymes may serve as therapeutic targets for the treatment of CVDs.^109^

In fact, the crosstalk between protein methylation and ubiquitination is frequently observed in the regulation of methyltransferases or ubiquitinase themselves, rather than just cardiovascular proteins themselves. For example, Methyltransferase like protein 13 (Mettl13) is a dual functional methyltransferase that targets the N-terminus of eukaryotic elongation factor 1A (eEF1A) and Lys55 for methylation, and this methylation can regulate the translation rate of specific codons.^112^^,^^113^ In HF, C-Cbl, as an E3 Ub ligase, may degrade SERCA2a through ubiquitination, thereby affecting the calcium processing and contractile function of myocardial cells. Mettl13 affects the function of C-Cbl or its interaction with SERCA2a through methylation, thereby inhibiting the ubiquitination and degradation of SERCA2a, maintaining its stability and cardiac contractile function. Mettl13 plays a protective role in ischemic HF by negatively regulating C-Cbl mediated SERCA2a ubiquitination, reducing SERCA2a degradation, and thus protecting cardiac contractile function.^114^ In addition, studies have reported that in the development of HF, HDAC3 modifies DNMT1 through deacetylation, inhibiting the ubiquitination-mediated degradation of DNMT1 and promoting its expression. DNMT1 inhibits the expression of SHP-1 through methylation in the promoter region. Inhibiting the ubiquitination of DNMT1 enables it to exert its methylation modification function.^90^ Moreover, research has confirmed that Ub-specific protease 22 (USP22) deubiquitinates lysine-specific demethylase 3A (KDM3A), causing nuclear translocation and thus exerting methylation modification function. Sevoflurane is a volatile anesthetic clinically used for cardiac surgery. Its cardioprotective effect in MIRI is mediated, in part, by upregulating USP22. USP22 then deubiquitinates and stabilizes KDM3A, which subsequently promotes protective transcriptional programs.^115^ On the other hand, methylation can also have an impact on ubiquitination. A case in point is the DNA methyl transferase 3B (DNMT3B). DNMT3B protein levels are reduced in the heart on a high-fat diet, which may affect gene expression and silencing. At the same time, the monoubiquitination level of histone H2A was significantly downregulated due to a high-fat diet, indicating the regulatory role of methylation in ubiquitination in CVDs.^116^ Intriguingly, Dot1L (disruptor of telomeric silencing 1-like) is the only known histone H3K79 methyltransferase.^117^ The blockade of Dot1L prevents receptor interacting protein kinase 1 (RIPK1) dependent apoptosis by increasing RIPK1 K63 ubiquitination and reducing the formation of RIPK1/aspase 8 complex, thereby reversing ischemia induced neuronal apoptosis.^118^

Overall, methylation-ubiquitination crosstalk in CVDs appears to occur more often through regulation of modifying enzymes and signaling proteins than through direct competition on a shared substrate residue.^119^ Defining these enzyme-centered interactions more precisely will be important for determining which pathways are mechanistically relevant and therapeutically tractable.

## Emerging roles of other PTMs in crosstalk with ubiquitination in CVDs

While the preceding sections focus on phosphorylation, SUMOylation, acetylation, and methylation, increasing evidence indicates that additional PTMs, including O-linked β-N-acetylglucosamine modification (O-GlcNAcylation), neddylation, and ADP-ribosylation/PARylation, also intersect with Ub-dependent protein turnover in CVDs. Compared with canonical phosphorylation-ubiquitination or acetylation-ubiquitination crosstalk, the cardiovascular evidence for these emerging PTMs remains less extensive. Nevertheless, several mechanistically defined studies have demonstrated that these modifications can regulate substrate ubiquitination, proteasomal degradation, or the activity of Ub E3 ligase complexes in vascular and cardiac disease.

### O-GlcNAcylation and ubiquitination

O-linked β-N-acetylglucosamine modification is a nutrient-sensitive and reversible PTM that attaches a single GlcNAc moiety to serine or threonine residues of intracellular proteins. Rather than acting uniformly as a stabilizing modification, O-GlcNAcylation can either promote or antagonize Ub-dependent degradation depending on the substrate, modified residue, and pathological context. A representative cardiovascular example is the anti-inflammatory protein A20, also known as TNFAIP3, in diabetic vascular disease. In human coronary endothelial cells and smooth muscle cells exposed to high glucose, as well as in diabetic vascular tissues, A20 protein expression was reduced despite preserved or increased mRNA levels. Mechanistically, high glucose promoted A20 O-GlcNAcylation, which was associated with increased A20 ubiquitination and proteasomal degradation. Loss of A20 enhanced pro-atherogenic signals, including RAGE expression and PKCβII phosphorylation, whereas inhibition of O-GlcNAcylation or proteasomal degradation, as well as A20 gene transfer, restored A20-dependent vascular protection and reduced accelerated atherosclerosis in diabetic ApoE-null mice.^120^ This study provides a direct example in which O-GlcNAcylation facilitates Ub-dependent degradation in cardiovascular pathology.

Conversely, O-GlcNAcylation can also stabilize disease-relevant proteins by suppressing their Ub-dependent turnover. In diabetes-associated coronary microvascular disease, excessive protein O-GlcNAcylation was linked to p53 accumulation in coronary endothelial cells, endothelial apoptosis, impaired coronary microvascular function, and cardiac dysfunction. Overexpression of O-GlcNAcase reduced protein O-GlcNAcylation and p53 abundance, thereby improving coronary microvascular and cardiac function in diabetic mice.^121^ Although the cardiovascular study mainly established the disease relevance of O-GlcNAc-driven p53 accumulation, the molecular basis is supported by earlier mechanistic work showing that O-GlcNAcylation of p53 at Ser149 reduces Thr155 phosphorylation and blocks Ub-dependent proteolysis, thereby stabilizing p53 (Figure 3A).^122^ More recently, HDAC4 was identified as another cardiovascular substrate whose O-GlcNAcylation affects protein stability. In a MI model, Ser455 O-GlcNAcylation stabilized HDAC4, whereas coenzyme Q10 promoted HDAC4 degradation by inhibiting this stabilizing O-GlcNAc modification, contributing to reduced cardiomyocyte injury and fibrotic activation.^123^ Together, these findings suggest that O-GlcNAcylation-ubiquitination crosstalk is context-dependent in CVDs: it may accelerate degradation of protective vascular proteins such as A20, while stabilizing injury-associated proteins such as p53 or HDAC4. This bidirectional crosstalk highlights the interplay between nutrient signaling and protein quality control in CVDs.

**Figure 3:**
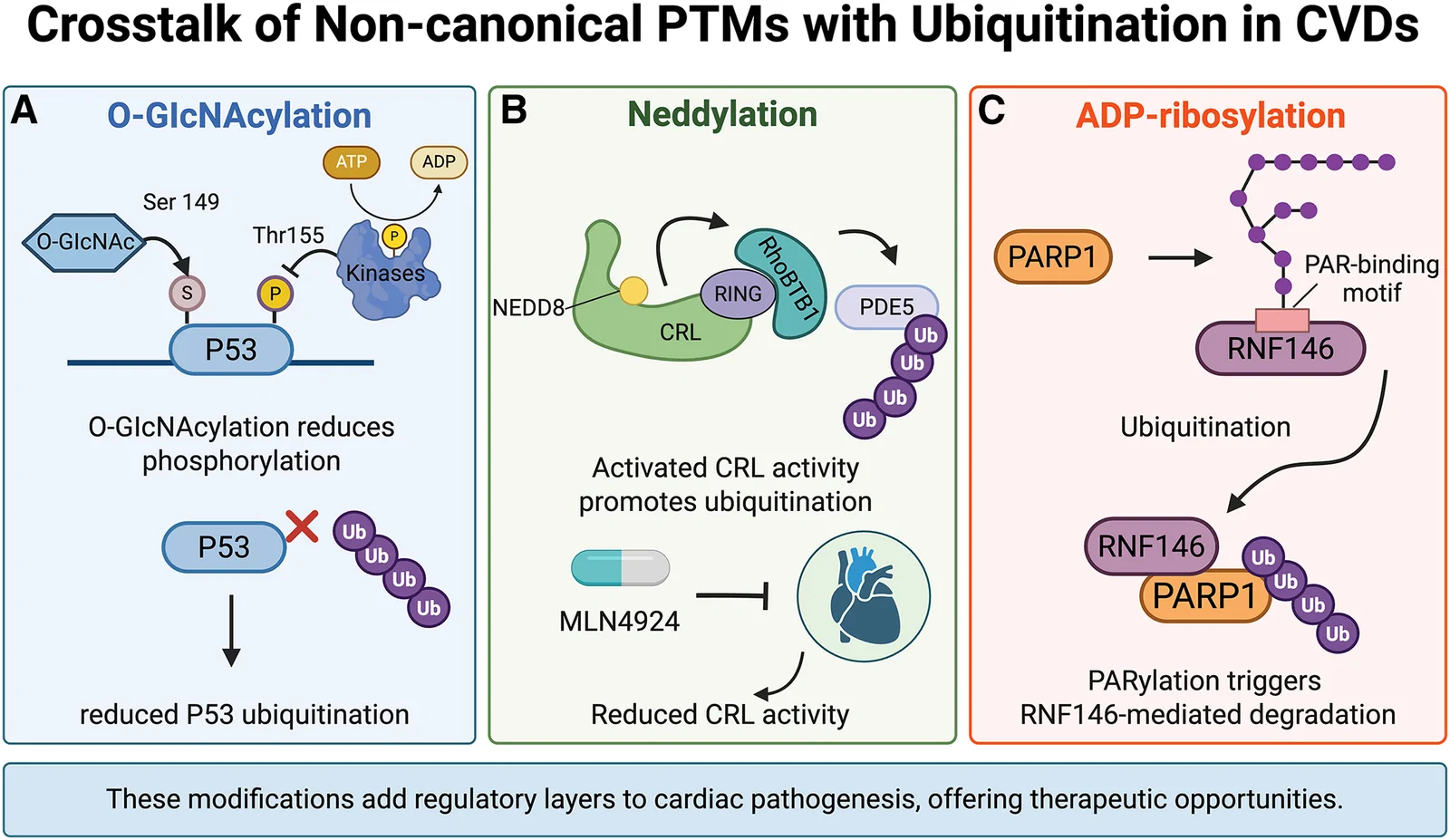
Emerging post-translational modifications and their crosstalk with ubiquitination in cardiovascular diseases This figure summarizes three additional PTMs that intersect with ubiquitination through distinct mechanisms. (A) O-GlcNAcylation. O-linked β-N-acetylglucosamine (O-GlcNAc) modification can either promote or inhibit ubiquitin-dependent degradation depending on the substrate. In diabetic vascular disease, O-GlcNAcylation of the anti-inflammatory protein A20 (TNFAIP3) enhances its ubiquitination and proteasomal degradation, accelerating atherosclerosis. Conversely, O-GlcNAcylation of p53 at Ser149 reduces its ubiquitination, stabilizing p53 and contributing to coronary microvascular dysfunction in diabetes. O-GlcNAcylation of HDAC4 (histone deacetylase 4) at Ser455 also stabilizes the protein, while coenzyme Q10 counteracts this modification. (B) Neddylation. The covalent conjugation of NEDD8 (neural precursor cell expressed developmentally down-regulated protein 8) to cullin scaffolds activates cullin-RING ubiquitin ligases (CRLs), which are the largest family of E3 ubiquitin ligases. In hypertension and arterial stiffness, the CRL3 adaptor RhoBTB1 (Rho-related BTB domain-containing protein 1) promotes ubiquitination and degradation of phosphodiesterase 5 (PDE5), preserving vascular relaxation. This process requires cullin neddylation. In cardiac hypertrophy, CAND1 (cullin-associated and neddylation-dissociated 1 protein) regulates CRL complex remodeling to enhance calcineurin ubiquitination and degradation, thereby attenuating hypertrophic remodeling. (C) ADP-ribosylation/PARylation. Poly(ADP-ribosyl)ation (PARylation) is catalyzed mainly by PARP1 (poly(ADP-ribose) polymerase 1) under oxidative stress. PAR chains serve as a recognition signal for the E3 ubiquitin ligase RNF146 (ring finger protein 146), which binds PAR via its WWE domain and becomes allosterically activated. RNF146 then ubiquitinates PARylated proteins, including PARP1 itself, targeting them for proteasomal degradation. This axis operates in myocardial ischemia/reperfusion injury and cardiac remodeling. Additionally, the E3 ligase WWP2 (WW domain-containing protein 2) directly ubiquitinates PARP1 independently of PARylation, providing another layer of regulation. A20, tumor necrosis factor alpha-induced protein 3 (TNFAIP3); CAND1, cullin-associated and neddylation-dissociated 1; CoQ10, coenzyme Q10; CRL, cullin-RING ubiquitin ligase; HDAC4, histone deacetylase 4; NEDD8, neural precursor cell expressed developmentally down-regulated protein 8; O-GlcNAc, O-linked β-N-acetylglucosamine; p53, tumor protein p53; PARP1, poly(ADP-ribose) polymerase 1; PARylation, poly(ADP-ribosyl)ation; PDE5, phosphodiesterase 5; RhoBTB1, Rho-related BTB domain-containing protein 1; RNF146, ring finger protein 146; WWP2, WW domain-containing protein 2.

### Neddylation and ubiquitination

Neddylation is the covalent conjugation of the Ub-like protein NEDD8 to target proteins through an E1-E2-E3 enzymatic cascade. The best-established function of neddylation is the activation of cullin-RING Ub ligases (CRLs), the largest family of Ub E3 ligases. NEDD8 conjugation to cullin scaffolds induces conformational rearrangements that enhance CRL-mediated substrate ubiquitination and degradation. Thus, neddylation does not simply parallel ubiquitination; instead, it often functions upstream of ubiquitination by licensing CRL activity.

A clear vascular example is the CRL3 adaptor RhoBTB1 in HT and arterial stiffness. RhoBTB1 promotes the ubiquitination and degradation of phosphodiesterase 5 (PDE5), thereby restraining PDE5 activity and preserving nitric oxide/cGMP-dependent vascular relaxation. Importantly, pharmacological inhibition of neddylation prevented PDE5 ubiquitination by the CRL3 complex, indicating that cullin neddylation is required for this ubiquitination event (Figure 3B). Loss of RhoBTB1 increased PDE5 activity, impaired vasorelaxation, and contributed to HT and arterial stiffness, whereas restoration of RhoBTB1 had vascular protective effects.^124^ This study provides a direct example of neddylation-dependent CRL activity controlling ubiquitination of a cardiovascular substrate.

Neddylation-related regulation of CRL function has also been implicated in cardiac hypertrophy and HF. Cullin-associated and neddylation-dissociated 1 protein (CAND1) regulates cullin complex remodeling and substrate receptor exchange. In pressure overload-induced cardiac hypertrophy, CAND1 exerted anti-hypertrophic effects partly by promoting assembly of the Cul1/atrogin-1/calcineurin complex, thereby enhancing calcineurin ubiquitination and degradation. CAND1 deficiency aggravated cardiac hypertrophy, whereas CAND1 overexpression attenuated hypertrophic remodeling and improved cardiac function.^125^ Although CAND1 itself is not a NEDD8-conjugated substrate, this case illustrates how the neddylation state and remodeling of cullin complexes can shape Ub-dependent degradation of hypertrophic signaling proteins. Therefore, neddylation-ubiquitination crosstalk in CVDs should be viewed as a regulatory layer controlling E3 ligase activity, substrate selection, and protein turnover, rather than as a simple one-substrate dual modification model.

Notably, the therapeutic implications of neddylation inhibition in CVDs remain context dependent. Although the NEDD8-activating enzyme inhibitor MLN4924/pevonedistat can suppress CRL activity, global inhibition of neddylation may affect cardiomyocyte proliferation, cardiac development, inflammatory signaling, and proteostasis. Thus, therapeutic targeting of this pathway will likely require disease-stage- and cell-type-specific strategies rather than broad, systemic blockade.^126^

### ADP-ribosylation and ubiquitination

Poly(ADP-ribosyl)ation is catalyzed mainly by PARP family enzymes, among which PARP1 is a major stress-responsive enzyme involved in DNA damage responses, inflammation, and cell death. PARP1 uses NAD^+^ to generate poly(ADP-ribose) chains on itself and other proteins. Excessive PARP1 activation is well recognized in myocardial I/R injury, where it contributes to NAD^+^/ATP depletion, mitochondrial dysfunction, and cell death. In addition to these metabolic effects, PARylation also provides a molecular signal for ubiquitination.

The best-characterized PARylation-dependent ubiquitination pathway involves RNF146. RNF146 contains a WWE domain that binds PAR chains and an RING domain that mediates Ub ligase activity. Structural and biochemical studies have shown that PAR binding allosterically activates RNF146, thereby coupling PARylated substrates to ubiquitination and proteasomal degradation.^127^ In cardiac myocytes, RNF146 directly interacts with PARP1. Upon oxidative stress-induced PARP1 activation, RNF146 translocates to the nucleus, promotes PARP1 nuclear export, and facilitates rapid degradation of PARP1 (Figure 3C). RNF146 overexpression protects cardiomyocytes against oxidant-induced injury, whereas RNF146 silencing aggravates PARP1-mediated cell injury. Importantly, in a mouse model of myocardial I/R injury, PARP1 and RNF146 degradation occurred early in the ischemic/reperfused myocardium and correlated with reduced free Ub levels, supporting the existence of a PARP1-RNF146-Ub-proteasome axis in cardiac injury.^128^

In addition to RNF146, PARP1 stability in cardiovascular remodeling can be regulated by other Ub E3 ligases. For example, WWP2 selectively targets PARP1 for ubiquitination and degradation in isoproterenol-induced cardiac remodeling. Myocardial WWP2 deficiency reduced PARP1 ubiquitination and exacerbated adverse remodeling, whereas PARP1 degradation mediated by WWP2 exerted protective effects.^23^ Although this WWP2 example is not necessarily PARylation-dependent, it reinforces the concept that controlled Ub-mediated PARP1 turnover is an important regulatory mechanism in cardiovascular injury and remodeling.

In summary, O-GlcNAcylation, neddylation, and ADP-ribosylation/PARylation expand the regulatory landscape of ubiquitination crosstalk in CVDs. O-GlcNAcylation can either promote or inhibit ubiquitin-dependent degradation in a substrate-specific manner; neddylation mainly controls ubiquitination through activation and remodeling of CRL E3 ligases; and PARylation can serve as a recognition signal for PAR-dependent ubiquitination, particularly through RNF146. However, compared with phosphorylation or acetylation, the cardiovascular evidence for these emerging PTM-ubiquitination axes remains relatively limited. Future studies using site-specific mutants, PTM-resolved proteomics, and cell-type-specific *in vivo* models are needed to distinguish causal PTM crosstalk from secondary stress responses and to evaluate their therapeutic potential in CVDs.

## Conclusion and perspectives

Advances in mass spectrometry, single-molecule imaging, and bioinformatics have expanded the study of ubiquitination and PTM crosstalk in CVDs.^12^^,^^38^^,^^111^^,^^129^^,^^130^ These approaches have increasingly revealed cooperative and antagonistic interactions among PTMs in cardiovascular disease.^79^^,^^99^ However, several important challenges remain. For example, the interactions between different PTMs have high dynamics and spatiotemporal specificity, and accurately simulating and detecting these interactions in *in vitro* and *in vivo* experiments is one of the current research challenges.^131^^,^^132^ In addition, due to the diverse and interwoven complex regulatory network of PTMs, how to accurately identify and intervene in specific PTMs for the treatment of CVDs is also an important direction for future research.^14^^,^^133^

A critical issue remains distinguishing between PTM crosstalk that is causally related to disease initiation versus changes that occur as secondary consequences of pathology. Many studies to date are correlative, identifying altered PTM levels in diseased tissues without functional validation. Future research must employ dynamic, time-resolved analyses and *in vivo* models with conditional knockouts of specific PTM enzymes to establish causality. This distinction is essential for translating PTM-based findings into prognostic biomarkers or therapeutic targets.

A better understanding of these interactions may facilitate the development of interventions targeting specific PTM enzymes, substrates, or modification sites.^134^ These strategies may include small-molecule inhibitors, gene editing techniques such as CRISPR-Cas9, antibody drugs, and cell-based therapies. Such approaches may allow disease-relevant PTM pathways to be modulated without broadly disrupting cellular protein homeostasis.^134^^,^^135^^,^^136^ In addition, the possibility of combination therapy can be explored to achieve more comprehensive and effective therapeutic effects by intervening in multiple PTMs simultaneously.

In summary, ubiquitination-centered PTM crosstalk represents an important layer of cardiovascular regulation. Defining when, where, and on which substrates these interactions occur will be essential for distinguishing causal mechanisms and for identifying therapeutic pathways (Table 1).

**Table 1:** Representative examples of crosstalk between ubiquitination and other post-translational modifications in cardiovascular diseases

Protein/target	PTM crosstalk	Mechanism of interaction	Cardiovascular context	Functional outcome	References
SERCA2a	SUMOylation + ubiquitination + acetylation	SUMOylation stabilizes SERCA2a, while ubiquitination promotes degradation; acetylation modulates activity	heart failure	regulates calcium handling and cardiac contractility	Ye et al.^21^; Kho et al.^69^; Han et al.^72^; Gorski et al.^79^
CaMKIIδ	phosphorylation + ubiquitination	deubiquitination enhances CaMKIIδ phosphorylation and activation	heart failure	promotes calcium mishandling and cardiac dysfunction	Xu et al.^49^
PARP1	acetylation + ubiquitination	competing PTMs on lysine residues regulate PARP1 stability	cardiac remodeling/IRI	controls cell death and metabolic stress	Zhang et al.^102^
p53	O-GlcNAcylation + ubiquitination	O-GlcNAcylation inhibits ubiquitin-dependent degradation, increasing stability	coronary microvascular disease	promotes endothelial apoptosis	Si et al.^121^; Yang et al.^122^
HDAC4	O-GlcNAcylation + ubiquitination	O-GlcNAcylation stabilizes HDAC4; inhibition promotes degradation	myocardial infarction	regulates cardiomyocyte injury and fibrosis	Xie et al.^123^
PDE5	neddylation (CRL3) + ubiquitination	cullin neddylation activates CRL3, promoting PDE5 ubiquitination	hypertension/vascular stiffness	maintains vascular relaxation	Mukohda et al.^124^
Calcineurin	neddylation (via CAND1) + ubiquitination	cullin complex remodeling enhances calcineurin degradation	cardiac hypertrophy	suppresses hypertrophic signaling	Li et al.^125^
PARP1	PARylation + ubiquitination (RNF146)	PAR binding activates RNF146 E3 ligase, inducing PARP1 degradation	ischemia-reperfusion injury	limits oxidative stress-induced damage	DaRosa et al.^127^; Gerö et al.^128^
PARP1	ubiquitination (WWP2)	E3 ligase-mediated ubiquitination controls PARP1 turnover	cardiac remodeling	reduces adverse remodeling	Zhang et al.^23^

SERCA2a, sarco/endoplasmic reticulum Ca^2+^-ATPase 2a; CaMKIIδ, Ca^2+^/calmodulin-dependent protein kinase II delta; PARP1, poly(ADP-ribose) polymerase 1; HDAC4, histone deacetylase 4; PDE5, phosphodiesterase 5; CRL3, cullin-RING ligase 3; CAND1, cullin-associated and neddylation-dissociated 1; RNF146, ring finger protein 146; WWP2, WW domain-containing E3 ubiquitin protein ligase 2; O-GlcNAcylation, O-linked β-N-acetylglucosamine modification; PARylation, poly(ADP-ribosyl)ation; IRI, ischemia-reperfusion injury.

## Acknowledgments

The figures were created with Biorender.com. This work was supported by the Youth Scientific Research Project of Wuxi Municipal Health Commission (Q202204).

## Author contributions

Y.Y. and Z.L. contributed equally to this work. They jointly conceived the review topic, conducted the literature search, and drafted the manuscript. T.X. supervised the project and created the visualizations. All authors critically revised the text, approved the final version, and agree to be accountable for all aspects of the work.

## Declaration of interests

The authors declare no competing interests.
